# Vergleich der sterilen Spendertomographie in der Hornhautbank mit der Tomographie des Transplantates nach perforierender Keratoplastik

**DOI:** 10.1007/s00347-020-01256-6

**Published:** 2020-11-30

**Authors:** A. Quintin, L. Hamon, S. Mäurer, A. Langenbucher, B. Seitz

**Affiliations:** 1 Klinik für Augenheilkunde, Universitätsklinikum des Saarlandes (UKS), Homburg/Saar, Deutschland; 2 Institut für Experimentelle Ophthalmologie, Universität des Saarlandes, Homburg/Saar, Deutschland

**Keywords:** Hornhauttransplantation, Optische Kohärenztomographie, Augenbank, Spenderhornhaut, Kunstlinsenberechnung, Corneal transplantation, Optical coherence tomography, Eye banking, Donor cornea, IOL power calculation

## Abstract

**Hintergrund:**

Seit 2018 verwenden wir die sterile Spendertomographie in der Hornhautbank routinemäßig, um refraktive Überraschungen nach Keratoplastik zu vermeiden. Ziel dieser Studie war es, Spendertomographieparameter mit Tomographieparametern des Transplantates nach perforierender Keratoplastik (PKP) zu vergleichen.

**Methoden:**

Diese Studie umfasste 193 Spendergewebe der Hornhautbank, die für eine PKP verwendet wurden (Transplantatdurchmesser 8,2 ± 0,7 mm). Messungen wurden mit dem optischen Kohärenztomographen des vorderen Augenabschnittes (VAA-OCT) Casia 2 (Tomey Corp., Nagoya, Japan) präoperativ sowie postoperativ nach 5 ± 4 Monaten bei liegenden Fäden und nach 22 ± 4 Monaten ohne Fäden durchgeführt. Post- und präoperative Werte wurden mithilfe des Wilcoxon-Rangsummentests verglichen.

**Ergebnisse:**

Postoperativ, bei liegenden (bzw. ohne) Fäden, war die Brechkraft (P) der Hornhautvorderfläche (v) im steilen Meridian (S) (PvS) unverändert (−0,2 dpt; *p* = 0,78) (um 2,7 dpt größer [*p* < 0,01]) und im flachen Meridian (F) (PvF) um 4,5 dpt (2,8 dpt) niedriger (*p* < 0,01) im Vergleich zu den Spendertomographiewerten. Der Astigmatismus (v) war um 4,3 dpt (5,4 dpt) größer (*p* < 0,01). An der Rückfläche (r) war PrS um 0,9 dpt (0,9 dpt) und PrF um 0,3 dpt (*p* < 0,01) (0,1 dpt [*p* = 0,42]) kleiner, während der Astigmatismus (r) um 0,7 dpt (0,9 dpt) größer war (*p* < 0,01). Die zentrale Hornhautdicke war um 55,7 μm (*p* < 0,01) (27,5 µm [*p* = 0,01]) kleiner. Die Gesamtbrechkraft ohne Fäden änderte sich nicht signifikant im Vergleich zur Hornhautbankmessung.

**Schlussfolgerung:**

Zentrale Hornhautdicke, Brechkraft (P) und Astigmatismus veränderten sich postoperativ im Vergleich zu den Spendertomographiewerten, mit Ausnahme von P im steilen Meridian der Vorderfläche bei liegenden Fäden sowie von P im flachen Meridian der Rückfläche nach Entfernung aller Fäden. Die Gesamtbrechkraft ohne Fäden ändert sich allerdings nicht signifikant. Diese Informationen könnten für eine Verbesserung der Kunstlinsenberechnung bei klassischer „Triple-Procedure“ von Bedeutung sein.

## Abstract (en)

**Background:**

Since 2018 we routinely use sterile donor tomography in the eye bank to avoid refractive surprises after keratoplasty. The purpose of this study was to compare donor tomography parameters measured with anterior segment optical coherence tomography (AS-OCT) with tomography parameters of the graft after penetrating keratoplasty (PKP).

**Methods:**

This study enrolled 193 eye bank donor corneal tissues used for PKP (graft diameter 8.2 ± 0.7 mm). Preoperative measurements were taken using the AS–OCT Casia 2 (Tomey, Nagoya, Japan). The measurements were repeated postoperatively after 5 ± 4 months with all sutures in place and after 22 ± 4 months after removal of all sutures. Preoperative and postoperative values were compared using a Wilcoxon signed-rank test.

**Results:**

Postoperatively, with all sutures in place (or without sutures), the dioptric power (*P*) at the anterior surface (a) of the cornea remained unchanged (–0.2 D, *p* = 0.78) (2.7 D bigger, *p* < 0.01) in the steep meridian (S) (PaS) and 4.5 D (2.8 D, *p* < 0.01) smaller in the flat meridian (F) (PaF) in comparison with the donor tomography values. The astigmatism (a) was 4.3 D (5.4 D, *p* < 0.01) bigger. At the posterior surface (*p*) the PpS was 0.9 D (0.9 D, *p* < 0.01) and PpF 0.3 D (*p* < 0.01) (0.1 D, *p* = 0.42) smaller, while the astigmatism (*p*) was 0.7 D (0.9 D) bigger (*p* < 0.01). The central corneal thickness was 55.7 μm (*p* < 0.01) (27.5 µm, *p* = 0.01) smaller. The total power of the transplant after removal of all sutures did not change significantly in comparison with the eye bank measurements.

**Conclusion:**

Central corneal thickness, dioptric power (*P*) and astigmatism all changed postoperatively compared to donor tomography values, except *P* at the steep meridian of the anterior corneal surface with all sutures in place and *P* at the flat meridian of the posterior corneal surface after removal of all sutures; however, the total power of the transplant after removal of all sutures did not change significantly. This information may be of value for IOL power calculation in the classical triple procedure.

Die Schwierigkeiten und Herausforderungen der Kunstlinsenberechnung bei klassischer „Triple-Procedure“ sind allgemein bekannt. Dieser Beitrag soll die Veränderung der Spenderhornhaut von der Lagerung in Kulturmedium II (entquellendes Kulturmedium, mit Dextran angereichert) bis nach der perforierenden Keratoplastik zeigen, um perspektivisch eine genauere Schätzung der Brechkraft der zu implantierenden Kunstlinse zu ermöglichen.

## Hintergrund und Fragestellung

Mehrere Autoren haben bereits über die Notwendigkeit eines erweiterten Screenings von Spenderhornhautgewebe zur besseren Erkennung von Hornhautanomalien wie Narben, Keratokonus, granulären Dystrophien oder operativen Veränderungen wie nach Laser-assistierter In-situ-Keratomileusis (LASIK) berichtet [9, 10, 15, 17].

Ein Konzept zur kontaktfreien tomographischen Messung und Charakterisierung von Hornhautgeweben wurde vorgestellt, um Spenderhornhäute in ihrem Zellkulturmedium auf Krümmungsanomalien (hohen Astigmatismus oder bereits bestehende Pathologien wie Keratokonus oder Zustand nach refraktiven Eingriffen) zu untersuchen [6]. Obwohl solche Spenderhornhäute zum Teil für eine perforierende oder anteriore lamelläre Keratoplastik kontraindiziert sind, können sie potenziell für posteriore lamelläre Eingriffe wie eine „Descemet membrane endothelial keratoplasty“ (DMEK) verwendet werden. Dieses Messkonzept mittels einer auf der optischen Kohärenztomographie (OCT) basierenden Technik wurde aufgrund der hohen Auflösung des optischen Kohärenztomographens durch mehrere Autoren als geeignete Screeningtechnologie von Spendergewebe in der Zellkultur belegt ohne Gefahr der Kontamination des Spendergewebes [1, 4, 6, 11]. Die Spendertomographie stellt daher eine objektive Entscheidungshilfe dar, um festzustellen, für welche Operationsverfahren die Spenderhornhaut geeignet ist, und sollte benutzt werden, um refraktive Überraschungen nach perforierender Keratoplastik zu vermeiden.

Ziel dieser Studie war es, Spendertomographieparameter mit Tomographieparametern des Transplantates nach perforierender Keratoplastik zu vergleichen bzw. den Unterschied der Parameter zwischen präoperativ bei Lagerung in der Hornhautbank und postoperativ zu untersuchen.

## Material und Methoden

### Präoperative Vorbereitung der Spenderhornhäute

Um eine vertrauenswürdige Vermessung der Spenderhornhäute (sklerokorneale Scheiben mit einem Durchmesser von 16 mm) zu ermöglichen, wurden diese nach routinemäßiger Entquellung gemessen. Dies geschieht durch ein mit 6 % Dextran T‑500 angereichertes und dadurch hypertones und entquellendes Kulturmedium (Medium II), in welchem die für eine Keratoplastik zugewiesenen Spenderhornhautgewebe typischerweise 1 bis 3 Tage vor der Transplantation umgebettet werden. Die Hornhautspendergewebe wurden in ihrer Zellkulturflasche (Primaria 25 cm^2^ Canted-Neck Cell Culture Flask, Corning Inc., Corning, NY, USA) mindestens 12 h nach der Umsetzung in Medium II gemessen [2].

Spenderhornhäute wurden nur bei einer Endothelzellzahl über 2000 Zellen/mm^2^ (bzw. über 1700 Zellen/mm^2^ für eine perforierende Keratoplastik à chaud), unauffälliger Endothelzellmorphologie, der Abwesenheit von Narben oder Keratokonuszeichen sowie bei unauffälliger mikrobiologischer Untersuchung des Zellkulturmediums durch verantwortliche Personen (gemäß TPL 20c) für eine perforierende Keratoplastik freigegeben.

### Messungen der Spenderhornhäute

**Figure 1:**
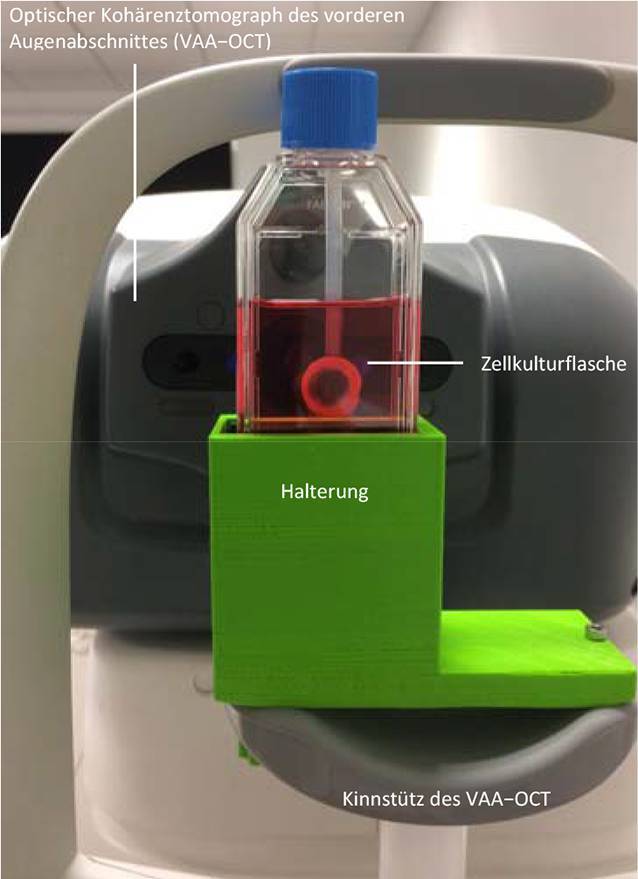

Messungen wurden mit dem optischen Kohärenztomographen des vorderen Augenabschnittes (VAA-OCT) Casia 2 (Tomey Corp., Nagoya, Japan) bei 193 Spenderhornhäuten durchgeführt. Dieses Gerät verwendet eine zentrale Wellenlänge von λ = 1310 nm und erlaubt eine Eindringtiefe von bis zu 13 mm in vivo. Daher sind auch Messungen von Spenderhornhäuten in ihrer Zellkulturflasche möglich, wobei ein lateraler Messbereich von ca. 7 mm Durchmesser erreicht wird, der hauptsächlich durch den Halter des Gewebes begrenzt ist [6]. Die Kulturflasche wurde in einer Halterung auf der Kinnstütze des OCT positioniert, die zuvor mit einem 3‑D-Drucker (Ultimaker 2Go, Ultimaker B.V., Geldermalsen, Niederlande) hergestellt wurde ([6]; Abb. 1).

Die gemessenen Rohdaten wurden in Matlab (The MathWorks Inc., Natick, Massachusetts, USA) importiert und analysiert. Nach der folgenden Bildvorverarbeitung gemäß der Strategie, wie bei Damian et al. dargestellt [1], wurde nach Entfernung von Artefakten, die von der Flaschenwand und dem Hornhauthalter herrühren können, eine Kantenerkennung der Vorder- und Rückfläche der Spenderhornhaut durchgeführt. Zunächst wurden die zentrale Dicke (CCT) sowie die Brechkraft (P) im flachen (F) und steilen (S) Meridian der Vorder- (v) und Rückfläche (r) der Spenderhornhaut bestimmt. Anschließend wurde daraus der Astigmatismus ermittelt. Die CCT wurde durch den Abstand zwischen der Vorder- und Rückfläche der Spenderhornhaut am Apex definiert. Messungen mit einer Pachymetrie >700 µm wurden ausgeschlossen.

### Chirurgische Technik

Spender- und Empfängertrepanation erfolgten mit dem gleichen Trepansystem, was eine Voraussetzung für kongruente Schnittflächen zwischen dem Spendergewebe und der Wirtshornhaut ist. Die Transplantatgröße (Transplantatdurchmesser 8,2 ± 0,7 mm) wurde individuell an die Hornhautgröße angepasst. Eine Excimerlasertrepanation [14] wurde für jede perforierende Keratoplastik mit einem Transplantatdurchmesser von 6,5–8,5 mm durchgeführt. Bei Transplantaten mit einem Durchmesser außerhalb dieser Grenzen wird eine mechanische Handtrepanation verwendet; 139 (72 %) Spenderhornhäute wurden mithilfe der doppelt fortlaufenden Kreuzstichnaht nach Hoffmann genäht und 54 (28 %) Spenderhornhäute mithilfe von Einzelknüpfnähten. Nach Entfernung der Lidsperrer und Haltefäden wurden am Ende der Operation via Keratoskopie mit einer handgehaltenen Placido-Scheibe offensichtliche Krümmungsanomalien des Transplantates durch geeignete Fadenumspannung korrigiert.

### Postoperative Messungen

Postoperativ wurden die Transplantate (*n* = 193) bei den Patienten mit demselben VAA-OCT Casia 2 nach 5 ± 4 Monaten bei liegenden Fäden (*n* = 172), nach 16 ± 3 Monaten nach Entfernung der Hälfte der Fäden (*n* = 42) sowie nach 22 ± 4 Monaten nach Entfernung aller Fäden (*n* = 27) gemessen. Die postoperative Bilderfassung dieser Patienten erfolgte in sitzender Position, nachdem ihr Kinn auf der Kinnstütze des VAA-OCT positioniert wurde. Postoperativ wurden die gleichen Parameter wie präoperativ untersucht.

Jede postoperative Messung wurde mindestens 6 Wochen nach Keratoplastik bzw. nach routinemäßiger Entfernung der Hornhautfäden durchgeführt. Postoperative Messungen wurden nur in Abwesenheit von Abstoßungs- und Immunreaktionen, bei einer Pachymetrie ≤700 µm sowie nur bei komplett epithelialisierten Hornhäuten ohne lockere Fäden eingeschlossen.

### Statistische Analyse

Eine statistische Analyse der oben genannten Parameter wurde mithilfe SPSS (IBM Corp., NY, USA) Version 20 durchgeführt. Die präoperativen Werte von jedem untersuchten Parameter der Spenderhornhaut wurden bei liegenden Fäden, nach Entfernung der ersten Hälfte der Fäden und nach Entfernung aller Fäden mittels eines Wilcoxon-Rangsummentests verglichen. Die berechneten Unterschiede bei liegenden Fäden wurden entsprechend der Nahttechnik mithilfe eines Mann-Whitney-U-Tests verglichen. Ergebniswerte werden als Mittelwert ± Standardabweichung (SD) ausgedrückt, sofern nicht anders angegeben. Ein *p*-Wert von <0,05 wurde als statistisch signifikant angesehen. Alle statistisch signifikanten *p*-Werte blieben auch nach Verwendung des Benjamini-Hochberg-Verfahrens für multiple Tests mit einer Falscherkennungsrate von 0,15 signifikant.

## Ergebnisse

Die Tab. 1 enthält die Werte der untersuchten Parameter postoperativ bei liegenden Fäden (*n* = 172), nach Entfernung der ersten Hälfte der Fäden (*n* = 42) und nach Entfernung aller Fäden (*n* = 27) sowie präoperativ nach routinemäßiger Messung der Spenderhornhäute (*n* = 193).

**Table 1:** 

			Organkultiviert (*n* = 193)	Mit liegenden Fäden (*n* = 172)	Nach Entfernung der 1. Hälfte der Fäden (*n* = 42)	Ohne Fäden (*n* = 27)
Vorderfläche	Brechkraft (dpt)	Steiler Meridian	45,6	45,4	47,6	47,9
		Flacher Meridian	44,2	39,7	42,4	41,3
	Astigmatismus (dpt)		1,4	5,7	5,2	6,5
Rückfläche	Brechkraft (dpt)	Steiler Meridian	−6,1	−7,1	−7,1	−7,0
		Flacher Meridian	−5,9	−6,2	−6,3	−6,0
	Astigmatismus (dpt)		0,2	0,9	0,8	1,1
Pachymetrie (µm)			584,6	528,3	519,5	561,4

Darstellung der Werte der untersuchten Parameter präoperativ in der Zellkulturflasche (*n* = 193) und postoperativ bei liegenden Fäden (*n* = 172), nach Entfernung der ersten Hälfte der Fäden (*n* = 42) sowie nach Entfernung aller Fäden (*n* = 27)

### Zustand mit liegenden Fäden

Postoperativ bei liegenden Fäden (Tab. 2a) zeigten alle Parameter einen signifikanten Unterschied (*p* < 0,01) im Vergleich zu dem präoperativen Wert mit Ausnahme der Brechkraft im steilen Meridian der Hornhautvorderfläche, die statistisch unverändert blieb (−0,2 dpt; *p* = 0,78). Im Vergleich zu präoperativ nahm die Brechkraft sowohl im steilen Meridian der Rückfläche (−0,9 dpt; *p* < 0,01) als auch im flachen Meridian der Vorder- (−4,5 dpt; *p* < 0,01) und Rückfläche (−0,3 dpt; *p* < 0,01) ab. Die Pachymetrie nahm ab (−55,7 μm; *p* < 0,01), und der Astigmatismus der Vorder- (+4,3 dpt; *p* < 0,01) und Rückfläche (+0,7 dpt; *p* < 0,01) der Hornhaut nahm bei liegenden Fäden im Vergleich zu den präoperativen Werten zu.

**Table 2:** 

*a. Bei liegenden Fäden (5* *±* *4 Monate postoperativ)*			
*n* = 172 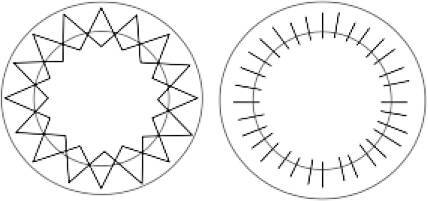			
		Vorderfläche	Rückfläche
Brechkraft (dpt)	Steiler Meridian	−0,2 ± 3,7 (*p* = 0,78)	−0,9 ± 0,7 (*p* < 0,01)
	Flacher Meridian	−4,5 ± 4,8 (*p* < 0,01)	−0,3 ± 0,6 (*p* < 0,01)
Astigmatismus (dpt)		+4,3 ± 4,0 (*p* < 0,01)	+0,7 ± 0,7 (*p* < 0,01)
Pachymetrie (µm)		−55,7 ± 57,2 (*p* < 0,01)	
*b. Nach Entfernung der ersten Hälfte der Fäden (16* *±* *3 Monate postoperativ)*			
*n* = 42 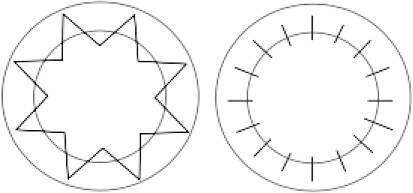			
		Vorderfläche	Rückfläche
Brechkraft (dpt)	Steiler Meridian	+2,3 ± 3,0 (*p* < 0,01)	−1,0 ± 0,4 (*p* < 0,01)
	Flacher Meridian	−1,7 ± 3,9 (*p* = 0,02)	−0,4 ± 0,5 (*p* < 0,01)
Astigmatismus (dpt)		+4,0 ± 3,5 (*p* < 0,01)	+0,6 ± 0,5 (*p* < 0,01)
Pachymetrie (µm)		−65,1 ± 89,1 (*p* < 0,01)	
*c. Ohne Fäden (22* *±* *4 Monate postoperativ)*			
*n* = 27 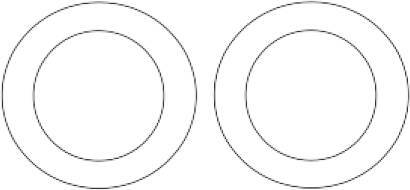			
		Vorderfläche	Rückfläche
Brechkraft (dpt)	Steiler Meridian	+2,7 ± 4,4 (*p* < 0,01)	−0,9 ± 0,8 (*p* < 0,01)
	Flacher Meridian	−2,8 ± 2,6 (*p* < 0,01)	−0,1 ± 0,5 (*p* = 0,42)
Astigmatismus (dpt)		+5,4 ± 5,0 (*p* < 0,01)	+0,9 ± 0,8 (*p* < 0,01)
Pachymetrie (µm)		−27,5 ± 56,6 (*p* = 0,01)	

### Zustand nach Entfernung der Hälfte der Fäden

Nach der Entfernung der ersten Hälfte der Fäden (Tab. 2b) änderten sich alle Parameter ausnahmslos (*p* < 0,05) im Vergleich zu präoperativ. Die Brechkraft im steilen Meridian nahm an der Vorderfläche zu (+2,3 dpt; *p* < 0,01) und an der Rückfläche der Hornhaut ab (−1,0 dpt; *p* < 0,01). Die Brechkraft im flachen Meridian nahm ihrerseits sowohl an der Vorder- (−1,7 dpt; *p* = 0,02) und Rückfläche (−0,4 dpt; *p* < 0,01) ab. Die Pachymetrie nach Entfernung der ersten Hälfte der Fäden nahm ab (−65,1 μm; *p* < 0,01) im Vergleich zu präoperativ im Gegensatz zum Astigmatismus der Vorder- (+4,0 dpt; *p* < 0,01) und Rückfläche (+0,6 dpt; *p* < 0,01) der Hornhaut.

### Zustand ohne Fäden

Nach der Entfernung der zweiten Hälfte der Fäden bzw. ohne Fäden (Tab. 2c) blieb nur die Brechkraft im flachen Meridian der Hornhautrückfläche (−0,1 dpt; *p* = 0,42) statistisch unverändert im Vergleich zu präoperativ. Die Brechkraft nahm im steilen Meridian der Vorderfläche zu (+2,7 dpt; *p* < 0,01) und im steilen Meridian der Rückfläche (−0,9 dpt; *p* < 0,01) sowie im flachen Meridian der Vorderfläche (−2,8 dpt; *p* < 0,01) ab. Die Pachymetrie nahm nach Entfernung aller Fäden ab (−27,5 µm; *p* = 0,01), und der Astigmatismus der Vorder- (+5,4 dpt; *p* < 0,01) und Rückfläche der Hornhaut (+0,9 dpt; *p* < 0,01) nahm im Vergleich zu den präoperativen Werten zu.

### Vergleich fortlaufende Nahttechnik und Einzelknüpfnähte

Die Tab. 3 zeigt den Vergleich des post- und präoperativen Unterschieds der untersuchten Parameter bei liegenden Fäden für die doppelt fortlaufende Kreuzstichnaht nach Hoffmann einerseits und für Einzelknüpfnähte andererseits. Alle Parameter zeigten bei liegenden Fäden einen statistisch signifikanten Unterschied (*p* < 0,05) abhängig von der Nahttechnik mit Ausnahme der Pachymetrie (−56,4 μm bei fortlaufenden Fäden; −54,1 μm bei Einzelknüpfnähten; *p* = 0,72). Bei liegenden Fäden war die Hornhautvorderfläche bei Einzelknüpfnähten signifikant flacher als bei fortlaufenden Fäden (im steilen Meridian −1,2 dpt bzw. +0,1 dpt, *p* = 0,03; im flachen Meridian −7,0 dpt bzw. −3,5 dpt, *p* < 0,01). Die Hornhautrückfläche war bei Einzelknüpfnähten im Vergleich zu fortlaufenden Fäden signifikant steiler geworden (im steilen Meridian −0,7 dpt bzw. −1,0 dpt, *p* < 0,01; im flachen Meridian +0,1 dpt bzw. −0,4 dpt, *p* < 0,01). Sowohl an der Vorder- als auch an der Rückfläche war der Astigmatismus bei liegenden Fäden bei Einzelknüpfnähten signifikant größer als bei fortlaufenden Fäden (an der Vorderfläche +5,8 dpt bzw. +3,6 dpt, *p* < 0,01; an der Rückfläche +0,9 dpt bzw. +0,6 dpt, *p* = 0,03).

**Table 3:** 

		Vorderfläche		Rückfläche	
		Fortlaufende Nähte (FL) 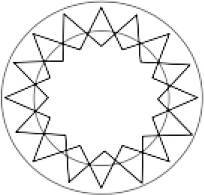	Einzelknüpfnähte (EKN) 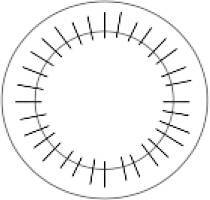	Fortlaufende Nähte (FL) 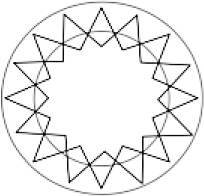	Einzelknüpfnähte (EKN) 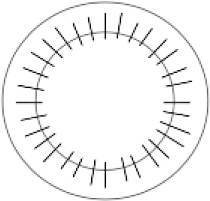
Brechkraft (dpt)	Steiler Meridian	+0,1	−1,2	−1,0	−0,7
		(*p* = 0,03)		(*p* < 0,01)	
	Flacher Meridian	−3,5	−7,0	−0,4	+0,1
		(*p* < 0,01)		(*p* < 0,01)	
Astigmatismus (dpt)		+3,6	+5,8	+0,6	+0,9
		(*p* < 0,01)		(*p* = 0,03)	
Pachymetrie (µm)		−56,4	−54,1	–	
		(*p* = 0,72)			

### Gesamtbrechkraft der Hornhaut

**Figure 2:**
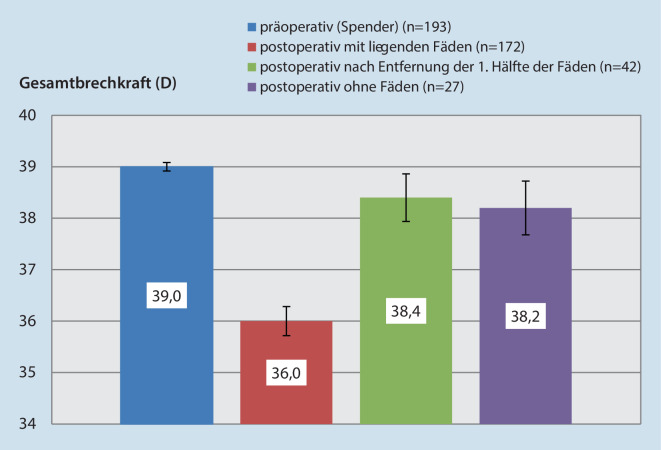

Die Abb. 2 enthält die prä- und postoperative Gesamtbrechkraft der Hornhaut, ermittelt nach der Gullstrand-Formel:

$$P_{\text{Gullstrand}}=P_{\text{Vorderfl{\"a}che}}+P_{\text{R{\"u}ckfl{\"a}che}}-\left[P_{\text{Vorderfl{\"a}che}}\times P_{\text{R{\"u}ckfl{\"a}che}}\times\frac{d}{n} \right]$$

Dabei bezeichnen $P_{\text{Gullstrand}}$ die gesamte Brechkraft und $P_{\text{Vorderfl{\"a}che}}$ und $P_{\text{R{\"u}ckfl{\"a}che}}$ die Brechkraft der Vorder- und Rückfläche, was dem Mittelwert der Werte aus dem steilen und flachen Meridian entspricht. $d$ repräsentiert die Hornhautdicke (in Metern), und $n$ den Brechungsindex der Hornhaut (1,376). Im Vergleich zu präoperativ (39,0 dpt) nahm die Gesamtbrechkraft der Hornhaut postoperativ bei liegenden Fäden signifikant ab (36,0 dpt; *p* < 0,01). Nach Entfernung der ersten Hälfte der Fäden (38,4 dpt; *p* = 0,53) sowie ohne Fäden (38,2 dpt; *p* = 0,39) konnte jedoch kein statistisch signifikanter Unterschied in der Gesamtbrechkraft mehr gezeigt werden im Vergleich zu dem präoperativen Wert (39,0 dpt).

## Diskussion

Heute beschränken sich die Erwartungen hinsichtlich der Ergebnisse nach einer perforierenden Keratoplastik (PKP) nicht nur auf die Klarheit des Transplantates. Ein klares Transplantat nach PKP mit hohem und/oder unregelmäßigem Astigmatismus, insbesondere in Kombination mit hoher Anisometropie, kann nicht als erfolgreich angesehen werden. Aus diesem Grund sollten Mikrochirurgen die bestmöglichen Maßnahmen zur Vermeidung von hohem oder unregelmäßigem Astigmatismus nach einer Keratoplastik ergreifen. Jeder einzelne Schritt, angefangen von der intraoperativen Trepanation, dem Durchmesser des Transplantates, der Nahttechnik bis hin zur Qualität der postoperativen Nachbehandlung, kann für das endgültige refraktive Ergebnis entscheidend sein ebenso wie die Auswahl des Spenders [3, 8, 12, 18, 19]. Sterile Spendertomographie in der Hornhautbank ist heute möglich [1, 4, 6, 11]. Spenderhornhäute mit einer Gesamtbrechkraft, die zu stark vom Durchschnittswert abweicht (z. B. außerhalb von ±3 Standardabweichungen vom Mittelwert), sollten idealerweise nicht für eine perforierende oder eine anteriore lamelläre Keratoplastik, sondern für eine posteriore lamelläre Keratoplastik verwendet werden, um postoperative refraktive Überraschungen zu vermeiden.

Die Größe des in der Studie benutzten Transplantates wurde individuell an die Hornhautgröße nach dem Prinzip „so groß wie möglich, so klein wie nötig“ angepasst, wobei größere Transplantate bezüglich des postoperativen Astigmatismus und kleinere Transplantate bezüglich der Immunologie von Vorteil sind [12]. Bei Transplantaten mit einem Durchmesser außerhalb der Grenzen 6,5–8,5 mm ist eine Excimerlasertrepanation mit dem zur Verfügung stehenden Lasersystem technisch nicht ausführbar, weshalb eine mechanische Handtrepanation verwendet wird, obwohl diese im Vergleich zu der Excimerlasertrepanation nachweislich mit einem höheren postoperativen Astigmatismus und niedrigerer postoperativer Sehschärfe verbunden ist [14, 16]. Als Nahttechnik wurde bei intakter Bowman-Lamelle die doppelt fortlaufende Kreuzstichnaht nach Hoffmann bevorzugt, da diese zu einer höheren topographischen Regularität und einer früheren visuellen Rehabilitation führt sowie mit einer geringeren Rate von Fadenlockerungen einhergeht [5]. Bei betroffener Bowman-Lamelle wurden im Gegensatz dazu typischerweise 24 Einzelknüpfnähte benutzt. Unsere Ergebnisse zeigen, dass Einzelknüpfnähte mit einer klinisch relevanten größeren Abflachung der Hornhautvorderfläche einhergehen, und bestätigen den größeren Astigmatismus bei Einzelknüpfnähten im Vergleich zu der doppelt fortlaufenden Kreuzstichnaht nach Hoffmann.

Darüber hinaus zeigen die oben gezeigten Ergebnisse, dass die Gesamtbrechkraft ohne Fäden nicht statistisch unterschiedlich ist im Vergleich zu der präoperativen Gesamtbrechkraft der Spenderhornhaut in der Zellkulturflasche. Diese Information könnte für eine Verbesserung der Kunstlinsenberechnung bei klassischer „Triple-Procedure“ von Bedeutung sein. Eine größere Anzahl von postoperativen Messungen ohne Fäden bei einer saubereren und homogeneren Patientengruppe ist jedoch erforderlich, um die gezeigten Ergebnisse zu bestätigen. Um in der Lage zu sein, eine Formel zustande zu bringen, welche die postoperative Gesamtbrechkraft abhängig von präoperativen Keratometriewerten vorhersagen kann, sollten andere Keratometrie- und Astigmatismus-beeinflussende Parameter wie Trepanationstechnik, Transplantatdurchmesser und Nahttechnik konstant gehalten werden.

Unsere Studie hat weitere Limitationen. Erstens verursachen die Lagerung und Anbringung der Hornhaut am Halter der Zellkulturflasche möglicherweise eine leichte Verformung des Spenderhornhautgewebes, wodurch die gemessene präoperative Geometrie nicht sicher mit In-situ-Bedingungen übereinstimmt. Zweitens stellen die prä- und postoperativen optischen Kohärenztomographien nicht automatisch die gleichen senkrechten Achsen dar, da die Gewebsorientierung zum Zeitpunkt der Operation nicht unbedingt beibehalten wurde [6].

Die „sterile Spendertomographie“ in der Hornhautbank wirft auch ein neues Licht auf die Möglichkeit der „Harmonisierung von Spender- und Empfängertomographie“ [7, 13]. Die mangelnde Übereinstimmung („Disharmonie“) zwischen Spender- und Empfängertomographie am Rande der Trepanation resultiert in unterschiedlichen Krümmungsverhalten zwischen Transplantat und Wirtshornhaut und ist deshalb eine der Ursachen für den persistierenden Restastigmatismus nach Fadenentfernung [13]. Durch die Aufeinanderabstimmung („Harmonisierung“) von Spender- und Empfängertomographie, bei welcher der steile und flache Meridian der Spenderhornhaut auf den flachen und steilen Meridian der Wirtshornhaut abgestimmt wird, kann der Restastigmatismus für ein gegebenes Spender-Empfänger-Paar möglicherweise minimiert werden.

Die Tomographie des Transplantates bietet ein objektives und steriles Screeningverfahren zur Identifizierung von Hornhautspendergeweben mit Krümmungsanomalien (hohem Astigmatismus, Keratokonus, Zustand nach refraktiven Eingriffen), um die Spenderauswahl weiter zu optimieren, und sollte daher, wenn möglich, zur Routine in der Hornhautbank eingesetzt werden.

## Fazit für die Praxis

- Die sterile und kontaktfreie Spendertomographie in der Hornhautbank ist heute möglich.
- Einzelknüpfnähte gehen bei liegenden Fäden mit einer klinisch relevanten größeren Abflachung der Hornhautvorderfläche und mit einem größeren Astigmatismus im Vergleich zu der doppelt fortlaufenden Kreuzstichnaht nach Hoffmann einher.
- Postoperativ nach Entfernung aller Fäden scheinen sich die Vorderflächenkrümmung sowie der Astigmatismus und die Pachymetrie im Vergleich zu den Spendertomographiewerten zu ändern. Die Brechkraft im flachen Meridian der Hornhautrückfläche zeigt jedoch keinen signifikanten Unterschied.
- Die postoperative Gesamtbrechkraft des Transplantates ist allerdings nach Entfernung aller Fäden vergleichbar mit der der Spendertomographie. Diese Information könnte für eine Verbesserung der Kunstlinsenberechnung bei klassischer „Triple-Procedure“ mittels Regressionsanalyse von Bedeutung sein.

## References

[1] Damian A, Seitz B, Langenbucher A, Eppig T (2017). Optical coherence tomography-based topography determination of corneal grafts in eye bank cultivation. J Biomed Opt.

[2] Hamon L, Daas L, Mäurer S, Weinstein I, Quintin A, Schulz K, Langenbucher A, Seitz B (2020) Thickness and curvature changes of human corneal grafts in dextran-containing organ culture medium before keratoplasty. Cornea. Online ahead of print 10.1097/ICO.0000000000002543 33290320

[3] Hoppenreijs VP, Van Rij G, Beekhuis WH, Rijneveld WJ, Rinkel-van Driel E (1993). Causes of high astigmatism after penetrating keratoplasty. Doc Ophthalmol.

[4] Janunts E, Langenbucher A, Seitz B (2016). In vitro corneal tomography of donor cornea using anterior segment OCT. Cornea.

[5] Jonas JB, Budde WM (1999). Loosening of single versus double running sutures in penetrating keratoplasty for keratoconus. Graefes Arch Clin Exp Ophthalmol.

[6] Mäurer S, Asi F, Rawer A, Damian A, Seitz B, Langenbucher A, Eppig T (2019). Konzept zur 3-D-Vermessung von Hornhautspendergewebe mithilfe eines klinischen OCT. Ophthalmologe.

[7] Mäurer S, Seitz B, Langenbucher A (2020). „Harmonization“ of donor and recipient tomography in corneal transplantation. Z. Med Phys.

[8] Naumann GOH (1995). Corneal transplantation in anterior segment diseases. The Bowman lecture (Number 56) Part II. Eye.

[9] Ousley PJ, Terry MA (2002). Objective screening methods for prior refractive surgery in donor tissue. Cornea.

[10] Ousley PJ, Terry MA (2002). Use of a portable topography machine for screening donor tissue for prior refractive surgery. Cornea.

[11] Priglinger SG, Neubauer AS, May CA, Alge CS, Wolf AH, Müller A, Ludwig K, Kampik A, Welge-Lüssen U (2003). Optical coherence tomography for the detection of laser in situ keratomileusis in donor corneas. Cornea.

[12] Seitz B, Langenbucher A, Küchle M, Naumann GO (2003). Impact of graft diameter on corneal power and the regularity of postkeratoplasty astigmatism before and after suture removal. Ophthalmology.

[13] Seitz B, Langenbucher A, Naumann GOH, Seiler T (2000). Astigmatismus bei Keratoplastik. Refraktive Chirurgie der Hornhaut.

[14] Seitz B, Langenbucher A, Kus MM, Küchle M, Naumann GO (1999). Nonmechanical corneal trephination with the excimer laser improves outcome after penetrating keratoplasty. Ophthalmology.

[15] Stoiber J, Ruckhofer J, Hitzl W, Grabner G (2001). Evaluation of donor tissue with a new videokeratoscope: the Keratron Scout. Cornea.

[16] Szentmáry N, Langenbucher A, Naumann GO, Seitz B (2006). Intra-individual variability of penetrating keratoplasty outcome after excimer laser versus motorized corneal trephination. J Refract Surg.

[17] Terry MA, Ousley PJ (1999). New screening methods for donor eye-bank eyes. Cornea.

[18] van Rij G, Cornell FM, Waring GO, Wilson LA, Beekhuis WH (1985). Postoperative astigmatism after central vs eccentric penetrating keratoplasties. Am J Ophthalmol.

[19] van Rij G, Waring GO (1998). Configuration of corneal trephine opening using five different trephines in human donor eyes. Arch Ophthalmol.

